# Adaptive experimentation and optimization in organic chemistry

**DOI:** 10.3762/bjoc.21.180

**Published:** 2025-11-03

**Authors:** Artur M Schweidtmann, Philippe Schwaller

**Affiliations:** 1 Process Intelligence Research Group, Department of Chemical Engineering, Delft University of Technology, Delft, Netherlands; 2 Laboratory of Artificial Chemical Intelligence (LIAC), Institute of Chemical Sciences and Engineering, École Polytechnique Fédérale de Lausanne (EPFL), Lausanne, Switzerland; 3 National Centre of Competence in Research (NCCR) Catalysis, École Polytechnique Fédérale de Lausanne (EPFL), Lausanne, Switzerland

The field of organic chemistry is undergoing a remarkable transformation. The convergence of laboratory automation and artificial intelligence is creating unprecedented opportunities for accelerating chemical discovery and optimization [1–2]. This thematic issue explores how adaptive experimentation, automation, and human–AI synergy are reshaping organic chemistry research.

Several key technological advances have enabled this transformation. High-throughput experimentation platforms can now rapidly test large numbers of reaction conditions [3]. Machine learning algorithms can process complex chemical data to identify promising directions [4]. Closed-loop systems can autonomously design, execute, and analyze experiments using machine learning optimization algorithms [5–6]. Together, these capabilities are dramatically increasing the speed and efficiency of chemical optimization with respect to economic and environmental objectives [7].

The contributions in this thematic issue showcase innovative approaches across multiple areas. Quijano Velasco et al. review recent advances in high-throughput automated chemical reaction platforms and machine learning algorithms for reaction optimization, showing how these approaches reduce experimentation time and human intervention [8]. They also discuss current limitations and outline future opportunities for this emerging field. Fralish and Reker demonstrate how active learning on molecular pairs can improve the identification of potent drug candidates [9]. Their ”ActiveDelta” method outperforms standard approaches while maintaining chemical diversity. Schmid et al. provide a comprehensive review of machine learning applications in enantioselective organocatalysis, highlighting both achievements and remaining challenges [10]. Guo et al. present an automated flow chemistry system for nitration reactions, combining kinetic modeling with experimental optimization [11].

However, the contributions in the thematic issue also reveal an important insight: while automation and AI are powerful tools, human chemical intuition remains invaluable. The work of Borup et al. on p*K*_a_ prediction illustrates how machine learning can complement rather than replace expert knowledge [12]. Their quantum chemistry-based workflow benefits from chemical understanding in selecting appropriate descriptors and validating predictions. Similarly, Chen and Li review how machine learning-guided optimization strategies are most effective when incorporating chemists’ expertise [13].

This theme of human–AI synergy emerges repeatedly. The computational design of asymmetric catalysts by Ferrer et al. demonstrates how AI can accelerate discovery while relying on chemical principles to guide the search space [14]. The most successful approaches combine the rapid exploration capabilities of AI with the deep understanding of experienced chemists.

Looking ahead, several key challenges and opportunities are apparent. Integrating prior knowledge and transfer learning between chemical domains remains difficult but promising. Improved methods for uncertainty quantification could help identify when human oversight is most needed. The development of more interpretable AI models would facilitate collaboration between human and machine intelligence.

The future likely lies not in fully autonomous systems but in thoughtfully designed frameworks that leverage both human and artificial intelligence. As the contributions in this thematic issue demonstrate, combining these complementary strengths can accelerate discovery while maintaining chemical insight and understanding.

We are grateful to all authors who have contributed to this thematic issue. Their work illustrates the tremendous progress in this field and the exciting opportunities ahead. As methods for adaptive experimentation continue to advance, maintaining focus on effective human–AI collaboration will be crucial for realizing the full potential of these technologies in organic chemistry.

This integration of automation, machine learning, and human expertise represents a new paradigm in chemical research. We hope this thematic issue provides valuable perspectives on current capabilities and future directions in this rapidly evolving field.

Artur M. Schweidtmann and Philippe Schwaller

Delft and Lausanne, October 2025

## Data Availability

Data sharing is not applicable as no new data was generated or analyzed in this study.
